# Antiviral Natural Products for Arbovirus Infections

**DOI:** 10.3390/molecules25122796

**Published:** 2020-06-17

**Authors:** Vanessa Shi Li Goh, Chee-Keng Mok, Justin Jang Hann Chu

**Affiliations:** 1 Laboratory of Molecular RNA Virology and Antiviral Strategies, Department of Microbiology and Immunology, Yong Loo Lin School of Medicine, National University of Singapore, Singapore 117545, Singapore; micvgsl@nus.edu.sg; 2 Infectious Disease Programme, Yong Loo Lin School of Medicine, National University of Singapore, Singapore 117597, Singapore; 3 Collaborative and Translation Unit for HFMD, Institute of Molecular and Cell Biology, Agency for Science, Technology and Research (A*STAR), Singapore 138673, Singapore

**Keywords:** antiviral, natural products, arbovirus

## Abstract

Over the course of the last 50 years, the emergence of several arboviruses have resulted in countless outbreaks globally. With a high proportion of infections occurring in tropical and subtropical regions where arthropods tend to be abundant, Asia in particular is a region that is heavily affected by arboviral diseases caused by dengue, Japanese encephalitis, West Nile, Zika, and chikungunya viruses. Major gaps in protection against the most significant emerging arboviruses remains as there are currently no antivirals available, and vaccines are only available for some. A potential source of antiviral compounds could be discovered in natural products—such as vegetables, fruits, flowers, herbal plants, marine organisms and microorganisms—from which various compounds have been documented to exhibit antiviral activities and are expected to have good tolerability and minimal side effects. Polyphenols and plant extracts have been extensively studied for their antiviral properties against arboviruses and have demonstrated promising results. With an abundance of natural products to screen for new antiviral compounds, it is highly optimistic that natural products will continue to play an important role in contributing to antiviral drug development and in reducing the global infection burden of arboviruses.

## 1. Introduction

Arthropod-borne viruses (arboviruses) are a significant cause of human morbidity and mortality worldwide. They are a large and diverse group of viruses that are transmitted from infected to susceptible hosts by the bite of an arthropod vector, predominantly mosquitoes and ticks. More than 500 arboviruses have been identified, with approximately 150 arboviruses known to cause disease in humans [1]. Most arboviruses causing human disease belong to four families: *Flaviviridae* (genus *Flavivirus*), *Togaviridae* (genus *Alphavirus)*, *Peribunyaviridae* (genus *Orthobunyavirus)*, and *Phenuiviridae* (genus *Phlebovirus*), with members of four other families, *Nairoviridae* (genus *Orthonairovirus*), *Orthomyxoviridae* (genus *Thogotovirus*), *Rhabdoviridae* (genus *Vesiculovirus*), and *Reoviridae* (genus *Orbivirus*) also contributing. Although arboviruses often cause large numbers of infections, the vast majority of cases are either asymptomatic or present as a flu-like illness. For this reason, the actual numbers of infection cases may be under-reported or misdiagnosed as other illnesses. Only a small proportion of cases progress to the more severe forms of arboviral disease that can be debilitating or potentially fatal, and usually affects young children or the elderly [2,3].

Over the course of the last 50 years, the emergence of several arboviruses—notably dengue, chikungunya, and Zika—have resulted in countless outbreaks globally, to which their geographical spread may be accelerated by rapid urbanization, deforestation, global warming, and increased international travel and trade [3,4,5,6]. These environmental and climate changes have brought people into more frequent contact with vectors, and have facilitated the expansion of vectors into new territories to cause disease [2,7,8]. With a high proportion of infections occurring in tropical and subtropical regions where arthropods tend to be abundant, Asia in particular is a region that is heavily affected by arboviral diseases [9]. The circulating arboviruses of public health concern in Asia can be classified into two groups: flaviviruses and alphaviruses. These include the dengue virus (DENV), Japanese encephalitis virus (JEV), West Nile virus (WNV), and Zika virus (ZIKV) which are flaviviruses, and chikungunya virus (CHIKV) which is an alphavirus (refer to Table 1) [10,11,12]. Severe clinical manifestations associated with each arbovirus mentioned herein have been observed. For instance, hemorrhagic fever in DENV infections, encephalitis in JEV and WNV infections; severe myalgia and arthralgia in CHIKV infections [1]. In the case of ZIKV infections, an increased occurrence of congenital microcephaly and Guillain–Barré syndrome was reported in ZIKV outbreak areas [13,14].

Despite advances in drug development, there are no antivirals available for the treatment of these rapidly spreading arboviruses. Current treatment available is primarily palliative, and solely serves to alleviate patients’ symptoms without any specific antiviral activity. Although the approval of some vaccines have been obtained for use in certain countries or are still undergoing development or clinical trials, it is still essential to develop treatments for people who are unable to receive the vaccine, and to care for unvaccinated infected patients [19]. Therefore, there is a desperate need for the discovery and development of potent and effective antivirals against arboviruses to reduce the infection burden which impacts millions across the globe.

A potential source of antiviral compounds could be discovered in natural products—such as vegetables, fruits, flowers, herbal plants, marine organisms, and microorganisms—from which various compounds have been documented to exhibit antiviral activity [20,21,22,23]. Screening natural products to discover novel antiviral compounds offers a head start on the drug discovery process, and could help to expedite the development of therapeutic and prophylactic treatment for arboviral diseases. This review article will summarize and discuss several studies on plant extracts, natural compounds including semi-synthetic derivatives, and synthetic compounds that have shown to demonstrate antiviral activity against DENV, JEV, WNV, ZIKV, and CHIKV that have been published in the last decade.

## 2. Flaviviruses

Flaviviruses (family *Flaviviridae*) are small enveloped, positive-sense single-stranded RNA viruses with a genome length of approximately 10–11 kb. The flaviviruses are a prominent group of arboviruses which include DENV, JEV, WNV, ZIKV, and many others such as yellow fever virus and hepatitis C virus (HCV).

### 2.1. Dengue Virus (DENV)

Transmitted by the *Aedes aegypti* and *Aedes albopticus* mosquitoes, DENV comprises of four antigenically related but distinct serotypes, DEN-1 to -4. According to the World Health Organization, dengue virus (DENV) is considered to be the most widespread arbovirus worldwide, with the disease endemic in more than 100 countries [24]. Of the 96 million cases of DENV infections that occur annually, 70% of the disease burden is contributed by Asia alone, followed by Latin America and Africa [25]. As the vast majority of cases are asymptomatic or mild, the actual numbers of dengue cases could be under-reported [26]. Symptoms of dengue infection include high fever (40 °C), severe headache, retro-orbital pain, and muscle and joint pain. In rare cases, severe dengue may occur with serious manifestations—including capillary leakage, fluid accumulation in tissue spaces, severe bleeding, and organ impairment—which can be potentially fatal [27]. Young children in particular are at greater risk of severe dengue as they may be less able to compensate for capillary leakage than adults [2].

Dengvaxia (also known as CYD-TDV) is the first tetravalent dengue vaccine to be licensed in 2015, but its performance is dependent on the serostatus of the receiving individual [28]. Clinical trials have shown that the vaccine is safe and efficacious in people who previously had a DENV infection (seropositive individuals); however, it confers a predisposition to severe dengue in those who experience their first natural dengue infection after vaccination (seronegative individuals) [27,29]. Therefore, it may not be suitable for everyone to receive the vaccine because of the risk involved. Currently, no specific therapeutic agent exists for dengue and treatment is mainly supportive.

### 2.2. Japanese Encephalitis Virus (JEV)

JEV is the leading cause of viral encephalitis in Asia with approximately 68,000 cases of infection estimated to occur annually [30]. *Culex* spp. mosquitoes are the vectors responsible for the transmission of JEV, which occurs mainly in rural agricultural areas, often associated with irrigated rice paddies [31]. The virus is maintained in a cycle between mosquitoes, pigs and/or wading water birds; humans are incidental hosts. Japanese encephalitis is primarily a disease of children, with ~75% of cases occurring in children aged below 15 years old [30]. It is reported that most adults in endemic regions have natural immunity after childhood infection; however, individuals of any age may be affected [30]. The vast majority of JEV infections are asymptomatic or present as a flu-like illness. JEV can effectively cross the blood–brain barrier (BBB) to cause acute encephalitis, with severe manifestations including high fever, headache, vomiting, seizures, disorientation, coma, and ultimately death [32,33]. The case-fatality rate can be as high as 30% among those with encephalitis, and 50% of survivors suffer permanent intellectual, behavioural, or neurological deficits [30,33,34].

Four main types of JE vaccines are currently in use, but the live attenuated SA14-14-2 vaccine is the most widely used vaccine in endemic countries, according to the WHO [35]. SA14-14-2 is administered in a two-dose regimen, and it appears to be safe and protective as no vaccine-induced severe effects have been observed [36]. Still, there is a risk of reversion of the live attenuated virus to virulence. Hence, an urgent need for a safe and effective cure for JEV infection and the development of a safer, single-dose vaccine is necessary as JEV continues to expand its activity into new territories.

### 2.3. West Nile Virus (WNV)

WNV is widely distributed in Africa, Europe, Australia, and Asia. Since its detection in New York City in 1999 [37], it continued to spread throughout the Western hemisphere—including other parts of USA, Canada, Mexico, and the Caribbean—resulting in significant morbidity and mortality [38]. WNV is maintained in nature in a mosquito-bird-mosquito transmission cycle, primarily involving *Culex* spp. mosquitoes and humans, horses, and other mammals are incidental hosts [39]. Most individuals infected with WNV are asymptomatic and ~20% of infected persons will experience clinical symptoms [40]. The majority of people with symptoms will develop West Nile fever, and only a small number will progress to severe West Nile neuroinvasive disease (WNND), which is potentially fatal (less than 1% of all infected people) [41]. Serious manifestations of WNND include encephalitis, meningitis, and acute flaccid paralysis [42]. Additionally, some patients suffer fatigue, muscle weakness, and persistent tremors, lasting weeks to years even after recovery [43,44,45]. Unfortunately, only supportive treatment is available for patients who suffer from severe and debilitating effects of WNND. To date, vaccines against WNV have been developed for horses [17,18], but there are no available vaccines for human use.

### 2.4. Zika Virus (ZIKV)

Discovered in 1947 in the Zika forest of Uganda [46], *Aedes* mosquitoes are the vectors responsible for ZIKV transmission. Since the 1950s, ZIKV had only been reported to be circulating in Africa and Southeast Asian countries including Indonesia, Thailand, Philippines, Malaysia, and Vietnam [13]. The first ZIKV outbreak recorded outside of Africa and Asia occurred in Yap Island, Federated States of Micronesia in 2007 [47]. This was followed by a larger epidemic in the French Polynesia in 2013–2014, and ZIKV subsequently spread to other Pacific islands [48,49]. In 2015, ZIKV emerged for the first time in the Americas, with a massive outbreak of infections occurring in Brazil [50,51].

Symptoms of ZIKV infection resembles that of dengue fever, which includes fever, headache, rashes, and joint and muscle pain. Although infections are usually mild or asymptomatic, neurological disorders have been associated with ZIKV infections. An increased occurrence of congenital microcephaly resulting from abnormal brain development, as well as Guillain–Barré syndrome (GBS) and acute myelitis in adults were reported in ZIKV outbreak areas [14,52,53,54,55]. Long-term consequences of microcephaly can range from mild developmental delays to severe motor and intellectual deficits, and often have poor prognosis [13,53]. In addition, patients who suffer from GBS experience a rapid onset of muscle weakness which may lead to incapability of walking, trouble swallowing and facial paralysis [13,14]. Although studies have shown that there is a strong association between these effects and ZIKV infection, its mechanism is still not well understood [14,52,53]. There is currently no specific antiviral treatment and vaccine to protect against the devastating consequences of ZIKV infection. Therefore, there is an urgent need for the development of therapeutic and prophylactic treatment for the management of ZIKV infection, especially in pregnant women and at-risk groups who live in ZIKV-endemic regions.

## 3. Alphaviruses

Alphaviruses (family *Togaviridae*) are small enveloped, positive-sense single-stranded RNA viruses with a genome length of approximately 11–12 kb. Arthropod-borne alphaviruses include CHIKV, Mayaro virus, Ross River virus, Semliki Forest virus, Sindbis virus, and O’nyong’nyong virus.

### 3.1. Chikungunya Virus (CHIKV)

First isolated from an outbreak in Tanzania in 1952, Chikungunya derives its name from the Tanzanian Makonde dialect that means “that which bends up”, referring to the posture of patients afflicted with extreme joint pain, which is a distinguishing trait of the illness [56,57,58]. Since 1952, CHIKV has caused a few sporadic outbreaks, mainly in Africa, Southeast Asia and India, many of them involving hundreds of thousands of people [58,59,60]. CHIKV only attracted worldwide attention when it caused an explosive outbreak in 2005–2006 on the French island of La Réunion (population: 770,000) infecting 40% of its inhabitants and resulting in more than 250 fatalities [61,62]. CHIKV is transmitted by *Aedes* mosquitoes and causes CHIK fever, which is accompanied by headache, maculopapular rash, back pain, myalgia, and polyarthralgia [57,63]. Complications of CHIKV infection include respiratory failure and meningoencephalitis [13]. Some patients have been reported to experience recurrent and persistent myalgia and arthralgia which last for weeks to months even after recovery, resulting in long-term disability [56,60,64]. However, there is currently no approved antiviral treatment or vaccine for CHIKV infection; treatment is usually symptomatic [65]. To date, CHIKV has been detected in more than 40 countries, including Indonesia, Thailand, Singapore, Cambodia, India, USA, Italy, and France [66,67,68,69,70].

### 3.2. Mayaro Virus

Mayaro virus (MAYV) is a neglected tropical arbovirus known to cause sporadic outbreaks in rural communities of South America, including Brazil, Peru, Venezuela, and Bolivia [71,72,73]. First isolated in 1954 in Trinidad, it is transmitted by forest-dwelling *Hemagogus* mosquitoes; however, the vector competence of the urban *Ae. aegypti* mosquito has been confirmed experimentally [74]. Symptoms of Mayaro fever include maculopapular rash, fever, and arthralgia [75]. Similar to CHIKV, persistent arthralgia in patients one year after symptom onset has been reported [75]. However, its symptoms may be clinically indistinguishable from dengue and chikungunya fever, hence leading to the misdiagnosis and underestimation of the number of MAYV infection cases [76,77]. Due to the potential of MAYV mimicking the epidemiological progression of CHIKV, it could pose a serious threat to public health systems in many countries [76]. In addition, the possibility of MAYV becoming further urbanized to spread to new territories mediated by *Ae. aegypti* and consequently causing large epidemics is worth noting. Therefore, increased surveillance and control plans must be considered to prepare for its possible emergence [78,79].

## 4. Natural Products as a Source of Antiviral Compounds

Medicinal plants have been used for millennia in the treatment and prevention against diseases and even until now, traditional medicine involving the use of herbal products are still widely practiced in Africa, Latin America, and China, and is gaining popularity in other countries including Australia and USA [80]. As the development of antivirals from synthetic molecules require a great amount of time and effort to design and validate from scratch, the discovery of antiviral compounds from natural products offers a more economical and simple alternative as they are easily accessible from nature [81,82,83]. Over the years, polyphenols—which are naturally occurring compounds found in fruits, vegetables, wine, and tea—have increasingly gained scientific interest as they have been reported to exhibit a wide range of pharmacological activities such as antiviral, anti-bacterial, antioxidant, anti-inflammatory, and anti-carcinogenic effects [84,85,86,87,88]. They are secondary metabolites of plants that are involved in protecting the plant from UV radiation, microbial infection, and defence against insects [89]. In food, polyphenols may contribute to the colour, flavour, odor, bitterness, astringency and oxidative stability [90]. Furthermore, they are expected to have minimal side effects as they form an integral part of the human diet.

Flavonoids are an important class of polyphenols with more than 4000 varieties identified in various plant species, and can be further divided into six subclasses: flavonols, flavones, flavanones, flavanols, anthocyanin, and isoflavones [89,90,91]. Regardless of the type of flavonoid, they are always characterized by a chromane ring bearing either a 2- or 3-phenyl ring which classifies them as a flavonoid, or an isoflavonoid, respectively [20]. The complexity of chemical structures that flavonoids offer may present potential broad-spectrum antiviral activities that can target various enzymes to disrupt the viral replication cycle [20,23,92,93]. In addition, not all flavonoids are absorbed with the same efficiency as its structural variation influences the rate of absorption, metabolism, and bioavailability in the body [94]. Hence, the antiviral effects of flavonoids are dependent on its bioavailability—the proportion of active ingredient that is digested, absorbed, and metabolized through normal pathways and become available at the site of action [89,90]. Therefore, in vitro results do not necessarily translate into efficacy in vivo and will require modifications to the compound to improve its bioavailability, so as to further clinical development against the target viruses. Strategies to overcome the poor systemic bioavailability of compounds will be discussed later. Numerous flavonoids, as well as plant extracts, have been reported for their antiviral activities against enveloped RNA viruses, including flaviviruses and alphaviruses (refer to Table 2 and Table 3). This review will highlight several interesting natural compounds and extracts which possess great antiviral potential.

### 4.1. Antiviral Natural Compounds

#### 4.1.1. Curcumin

Curcumin is a yellow pigment present in turmeric, a spice widely used throughout the world, and in traditional Asian and African medicine to treat a wide range of illnesses for over 4000 years. It has been shown to exhibit antioxidant, anti-inflammatory, anti-microbial, and anti-carcinogenic effects [94,116]. Moreover, the safety, tolerability, and non-toxicity of curcumin at high doses are well established in various human and animal models [116,117,118,119]. Besides being able to inhibit DENV, JEV, ZIKV, and CHIKV [97,98,99], curcumin has been reported to exhibit broad-spectrum antiviral activity against several enveloped viruses including PR8 influenza virus, vaccinia virus, pseudorabies virus, Newcastle disease virus [98], HCV [120], and human immunodeficiency virus (HIV) [121]. In contrast, the non-enveloped enterovirus-A71 (EV-A71) remained unaffected by curcumin treatment [98]. In general, the specific mechanisms by which curcumin exerts its antiviral effects against arboviruses is not well understood (refer to Table 2), but it has been proposed that the compound inhibits virus attachment to host cells [99].

Although curcumin is safe and efficacious, it is not an approved therapeutic agent because of its low bioavailability, poor aqueous solubility, and complications with drug delivery, which are challenges that have yet to be overcome [94,116]. To improve upon curcumin’s stability at plasma pH, Balasubramanian and co-workers (2019) generated four synthetic monoketone analogues of curcumin (curcuminoids) and tested them for their anti-DENV activity [97]. Three out of four synthetic curcuminoids—namely the acyclic, cyclopentanone, and cyclohexanone analogues of curcumin—were shown to possess better antiviral activities with improved stability as compared to native curcumin [97]. Modifications upon the chemical structure is one of the many strategies than can be employed to improve upon the bioavailability of curcumin [116].

#### 4.1.2. Epigallocatechin Gallate (EGCG)

Epigallocatechin gallate (EGCG) is a flavonoid abundant in green tea and has shown to possess potent antiviral activity against HIV, influenza virus, herpes simplex virus (HSV), and HCV [92,101,122,123]. A direct virucidal effect of EGCG was observed against DENV, CHIKV, WNV, and ZIKV, but its specific mechanism is not well understood (refer to Table 2) [92,100,101]. Three studies have collectively suggested that EGCG exerts its antiviral effect at the early stages of viral replication, possibly by binding directly to the viral particle to inhibit host cell attachment [92,100,101]. Carneiro and colleagues (2016) suggested that the antiviral mechanism by which EGCG exerts on ZIKV (and possibly other arboviruses) could be the same as HIV, as reported in a previous study [123]. In that study, it was established that EGCG could destroy and destabilize the viral envelope phospholipids, thus leading to virus destruction [123]. Despite being a natural compound, EGCG can be toxic to various cell lines at higher concentrations, but is regarded to be safe for use in healthy individuals [101,124]. In addition, a study in rats showed that EGCG can easily cross the placental barrier and spread to foetal tissues such as the brain, eyes, and heart [125]. Together, these studies show that EGCG is a potential antiviral candidate capable of inhibiting several arboviruses, and would be especially helpful to treat or prevent neuroinvasive arboviruses like JEV, WNV, and ZIKV.

#### 4.1.3. Pinocembrin

Pinocembrin is a flavanone abundant in propolis, honey, tea, and red wine. Widely studied for its role in neurodegenerative diseases and ischemic stroke, it has neuroprotective, antioxidant, anti-inflammatory, as well as anti-microbial effects [126,127]. It has been shown to be able to cross the BBB because of its low molecular weight and good liposolubility in an in vitro model using cultured rat brain microvascular endothelial cells (BMECs) [127,128]. Recently, Lee and colleagues (2019) reported that pinocembrin is able to inhibit ZIKV infection in human placental JEG-3 and human hepatoma Huh7 cell lines. The study determined that pinocembrin acts on post-entry processes of the ZIKV replication cycle but its exact mechanism remains to be studied [107]. The broad-spectrum antiviral activity of pinocembrin was also investigated in the same study using DEN-2, CHIKV, and EV-A71; significant inhibition against DEN-2 and CHIKV was demonstrated [107].

A phase I clinical trial for pinocembrin as a new neuroprotective agent reported no adverse effects when 120 mg/day of pinocembrin was administered intravenously to 58 healthy adults for 5 days, which suggests that pinocembrin is safe and well-tolerated [129]. In regards to using pinocembrin as an antiviral drug, more work needs to be done to validate its ability to reduce ZIKV-associated symptoms and neurodegenerative effects as well as to evaluate its safety for use in pregnant women [126].

#### 4.1.4. Quinine/Quinine Sulfate

Quinine is a natural compound extracted from the bark of the Cinchona tree and is an important anti-malarial agent which has been used to treat malaria from as early as the 1600s [130]. It was shown to exert antiviral activity on HSV [131], influenza [132], and DENV [109]. A study sought to repurpose four drugs as anti-DENV drug candidates by investigating their antiviral activity in all four serotypes of DENV [109]. Drug repurposing is a strategy which uses established drugs to treat diseases that lack specific therapeutic treatments as it is a faster and more cost-effective approach compared to de novo drug discovery and development [133]. Of the four drugs examined, quinine markedly decreased DENV replication by inhibiting DENV RNA replication, protein synthesis, and virus production by targeting host factors; however, its specific antiviral mechanism could not be determined [109]. As quinine is reported to act on host cells rather than directly upon the virion, resistance to the compound could be minimized [131].

The effects of quinine in the human body is well studied as it has been in use for years, primarily for the treatment of malaria. It has been reported that quinine produces mild to severe side effects at therapeutic concentrations in malaria treatment [130]. Mild symptoms include headache, nausea, and tinnitus; while more severe side effects include vertigo, vomiting, loss of vision, and marked auditory loss [130]. Identifying quinine as an antiviral drug is a good starting point, but more evaluation and calibration to optimize the dose and duration of treatment to minimize prolonged exposure and its associated side effects must be performed before it can be prescribed to treat viral infections.

#### 4.1.5. Other Potential Antiviral Compounds against Arboviruses

Other natural compounds that have been reported to exhibit antiviral activities, as well as the assays performed to determine the proposed mode of action have been summarized in Table 2. An overview of the compounds that can inhibit the different arboviruses is provided in Table 4.

### 4.2. Antiviral Plant Extracts

#### 4.2.1. *Psiloxylon mauritianum* Extract

An indigenous medicinal plant ubiquitous in the Reunion and Mauritius Islands, *Psiloxylon mauritianum* is widely used in traditional medicine among the local people for dysentery and common infectious diseases [113]. Given the high content of polyphenols present in *P. mauritianum* extract, a study evaluated its ability to inhibit ZIKV and DENV infections [113]. The data showed that *P. mauritianum* extract was able to inhibit ZIKV attachment to the host cell surface, and was able to exert antiviral activity against four DENV serotypes [113]. The plant extract displayed low toxicity and did not exert a genotoxic effect on human primary cell lines tested [113]. However, more work needs to be done to identify the active compounds responsible for antiviral activity from the plant extract and to investigate its potential to be an anti-flaviviral agent.

#### 4.2.2. Silymarin Complex

Silymarin is a complex extracted from milk thistle (*Silybum marianum*) seeds, and its major active compound is silybin [134]. Due to its antioxidant, anti-inflammatory, antifibrotic, and hepatoprotective activities, it is used as a supportive treatment for chronic liver diseases, hepatocellular carcinoma, and liver cirrhosis [134]. In a two-year toxicity study of silymarin conducted in mice, no adverse side effects were reported [135]. Even in humans, it is a well-tolerated compound after prolonged and high dosage use, with the most common side effects being headache and itching [136]. Silymarin was shown to inhibit HCV both in vitro and in vivo by inhibiting viral entry, RNA synthesis, and viral protein expression (refer to Table 3) [137]. In a study conducted by Lani and co-workers (2015), silymarin demonstrated significant in vitro antiviral activity against CHIKV by suppressing post-entry stages of the CHIKV replication cycle and inhibit CHIKV-induced cell death [114]. Additionally, silymarin has recently been reported to be able to inhibit MAYV, which is an alphavirus like CHIKV, by suppressing MAYV replication and attenuating MAYV-induced oxidative stress caused by MAYV infection [115]. This suggests that there could be potential in silymarin to be a broad-spectrum antiviral drug, but its activity against other viruses will need to be investigated further.

#### 4.2.3. Other Potential Antiviral Plant Extracts against Arboviruses

Table 3 lists two other plant extracts that have been reported to exhibit antiviral activity against arboviruses, as well as the assays done to determine the proposed mode of action. *Aphloia theiformis*, an edible indigenous plant from La Réunion, as well as mushroom extracts derived from each of the four culinary and medicinal mushrooms, *Lignosus rhinocerotis, Pleurotus giganteus, Hericium erinaceus* and *Schizophyllum commune*. An overview of the plant extracts which can inhibit the different arboviruses is provided in Table 5.

## 5. Conclusions

To address the current lack of antivirals against arboviruses, great efforts to search for potential antiviral compounds against arboviruses have been made by applying various approaches, from drug repurposing to the screening of different bioactive compound libraries, as well as natural products. In particular, polyphenols—which are naturally occurring compounds present in a wide array of fruits, vegetables, and plant parts (roots, bark, leaves and flowers)—have increasingly gained medicinal interest as they have shown to possess numerous health benefits and broad-spectrum antiviral activities in various studies. Screening natural products to discover novel antivirals offer a more time-efficient and economical alternative to traditional drug discovery processes that involve developing drug candidates from scratch, which can be inefficient and costly. In addition, the chemical structures of the novel compounds identified to exhibit antiviral activity could be utilized as a scaffold for designing semi-synthetic or synthetic drug derivatives so as to optimise its stability, or to enhance its antiviral potency. By developing antivirals against arboviruses, therapeutic treatment can be made available to infected patients to accelerate viral clearance and to reduce disease severity [138]. Other applications for anti-arboviral drug development include prophylactic or early post-exposure prophylactic treatment for people living in arbovirus endemic areas where vaccines against the arboviruses are not yet available, or for travellers visiting countries with ongoing outbreaks [138].

Many antiviral candidates have been found to inhibit viral replication using in vitro approaches, and only a few have been tested in vivo. This indicates the difficulty in translating experimental results to humans, and in completing the entire drug development process [139]. Nevertheless, careful evaluation must be conducted to understand the efficacy, tolerability, and safety of the drug by observing for any adverse side effects, or side effects associated with long-term exposure before using them in clinical practice. The target populations for receiving antiviral therapy will include children, elderly, and patients with pre-existing conditions, as well as pregnant women (risk of ZIKV infection) living in endemic areas, as they are especially vulnerable to the severe manifestations of arboviral diseases. In addition, further work is required to uncover the inhibition mechanisms of the antiviral candidates in order to elucidate the host or viral receptors involved in suppressing infection effectively, as well as to examine the possibility of combination therapies involving other natural compounds or established antivirals.

The low bioavailability of polyphenols in the body is one of several factors which hinder the development of antiviral drugs from natural compounds. It can be attributed to inefficient absorption in the intestine, poor water solubility, high metabolism rate, rapid systemic elimination, and remains a challenge faced by many [97,101,108,114,137]. Strategies to enhance the bioavailability of compounds have been employed based on changes in drug formulation, or modification to the chemical structure. Nanoparticle, micelle, and liposome-based drug formulations which encapsulates the drug in the particles have been successful in improving bioavailability [140,141,142,143,144]. Due to their small size and permeability, nanoparticles can improve delivery to membrane barriers, while micelles and liposomes can counter hydrophobicity as they can carry both hydrophilic and hydrophobic molecules [94,116]. In addition, modifications to the chemical structure could also improve bioavailability by adding complexes that are highly soluble in water to increase aqueous solubility and absorption in the gastrointestinal tract, or by the removal of chemical moieties to enhance stability of the molecule [94,97,145]. For instance, curcumin A, a curcumin analogue was synthesized by removing the β-diketone moiety from curcumin to improve stability as its presence renders it prone to decomposition at physiological pH [145]. Curcumin A was reported to withstand rapid degradation while retaining curcumin’s antiviral activity against HIV-1 infection in cultured lymphoblastoid T cells and peripheral blood mononuclear cells [145]. It was demonstrated to have improved stability in serum for up to 24 h as compared to curcumin, which is degraded by half within 10 h [145].

Natural compounds are expected to have good tolerability and minimal side effects, which are desirable traits for therapeutic and prophylactic treatment. In addition to vaccine development, drugs for prophylaxis, post-exposure prophylaxis, and treatment should be developed to prevent and mitigate the severity of arboviral diseases. As natural products provide a rich source of chemical diversity and pharmacological activities, it could potentially limit antiviral resistance, and also offer flexibility of use in other treatments including cancer, cardiovascular diseases, neurodegenerative diseases, and diabetes [91,126,146]. With an abundance of natural products to screen for new antiviral compounds, it is highly optimistic that natural products will continue to play an important role in contributing to antiviral drug development, and in reducing the infection burden that impacts millions globally.

## Figures and Tables

**Table 1:** List of common arboviruses circulating in Asia

Virus	Family/Genus	Transmission Vectors	Symptoms	Treatment Available?	Vaccine Available?
DENV	*Flaviviridae* (genus *Flavivirus*)	*Aedes aegypti* and *Aedes albopticus*	Fever, hemorrhagic fever.	No	Yes [15]
JEV	*Flaviviridae* (genus *Flavivirus*)	*Culex* spp.	Fever, headache, seizures, encephalitis.	No	Yes [16]
WNV	*Flaviviridae* (genus *Flavivirus*)	*Culex* spp.	Fever, muscle weakness, encephalitis, meningitis.	No	Not for humans. Vaccines for horses are available [17,18].
ZIKV	*Flaviviridae* (genus *Flavivirus*)	*Aedes* spp.	Fever, arthralgia, and myalgia. Neurological manifestations.	No	No
CHIKV	*Togaviridae* (genus *Alphavirus*)	*Aedes* spp.	Fever, arthralgia, and myalgia.	No	No

**Table 2:** List of natural compounds shown to exhibit antiviral properties against arboviruses (DENV, JEV, WNV, ZIKV, and CHIKV)

S/N	Compound Name/Chemical Structure	Source	Virus(es) Affected	Proposed Mode of Inhibition	Assay Used	Ref.
1	Baicalein 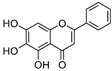	Roots of *Scutellaria baicalensis* and *Scutellaria lateriflora*	CHIKV	- Inhibits viral attachment to host cells - Has potent virucidal activity against extracellular viral particles	- Time-of-addition assay, inactivation assay	[95]
			JEV	- Possibly inhibits virus entry to cells - Has potent virucidal activity against extracellular viral particles	- Foci forming unit reduction assay, qRT-PCR	[83]
2	Baicalin (main metabolite of baicalein) 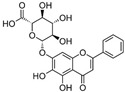	Roots of *Scutellaria baicalensis* and *Scutellaria lateriflora*	DEN-2	- Interferes and inhibits DENV-2 in vitro replication at various stages of the virus replication cycle	- Foci reduction assay, virus yield reduction assay	[96]
3	Curcumin 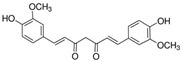	*Curcuma longa* (turmeric)	DEN-2	- Three synthesized monoketone analogues of curcumin were shown to have better antiviral activity than curcumin - Possibly inhibits virus by targeting host pathway essential for viral replication	- Plaque assay	[97]
				- Virucidal activity on enveloped viruses	- Plaque reduction assay	[98]
			CHIKV	- Inhibits viral attachment to host cells	- Time-of-addition assay	[99]
			ZIKV JEV	- Inhibits viral attachment to host cells	- Time-of-addition assay	[99]
				- Virucidal activity on enveloped viruses	- Plaque reduction assay	[98]
4	Delphinidin 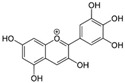	Pigment found in various flowers and fruits	DEN-1 to -4	- Virucidal activity observed	- Plaque assay	[92]
			WNV	- Affects early stages of viral replication cycle - Virucidal activity observed	- Plaque assay	[92]
			ZIKV	- Virucidal activity observed	- Plaque assay	[92]
5	Epigallocatechin gallate (EGCG) 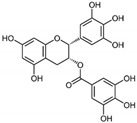	Leaves of *Camellia sinensis* (green tea)	DEN-1 to -4	- Virucidal activity observed	- Plaque assay	[92]
			CHIKV	- Possibly inhibits attachment to host cells	- Pseudotyping of lentiviral vectors with CHIKV glycoprotein and transduced HEK 293T cells	[100]
			WNV	- Affects early stages of viral replication cycle - Virucidal activity observed	- Plaque assay	[92]
			ZIKV	- Possibly inhibits attachment to host cells	- Pre-treatment assay	[101]
				- Virucidal activity observed	- Plaque assay	[92]
6	Fisetin 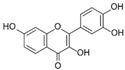	Pigment found in various flowers and fruits	CHIKV	- Inhibits early stages of viral replication	- Time-of-addition assay	[95]
7	Harringtonine 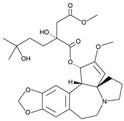	*Cephalotaxus harringtonia* (Japanese plum yew)	CHIKV	- Possibly by inhibiting viral protein synthesis	- Time-of-addition assay, qRT-PCR, SDS-PAGE, western blot	[102]
8	Honokiol 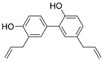	Bark or seed cones of Magnolia tree	DEN-2	- Inhibits viral entry to the host cells and suppresses in vitro viral replication	- Pre-treatment assay, viral yield reduction, and fluorescence focus formation assay	[103]
9	Isoquercitrin 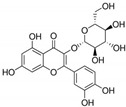	Various plants, including leaves of *Annona squamosa* (sugar apple) and *Camellia sinensis* (green tea)	ZIKV	- Inhibits viral entry to the host cell	- Time-of-addition assay	[104]
10	Kaempferol 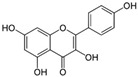	Various plant sources including tea, broccoli, grapefruit and apples	JEV	- Viral inactivation through binding to JEV frame-shift RNA	- qRT-PCR	[105]
11	Naringenin 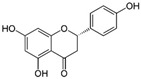	Citrus fruits such as grapefruit, bergamot, and tomatoes	DEN-1 to -4	- Impairs DENV replication cycle	- Time-of-addition assay	[106]
12	Pinocembrin 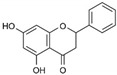	Honey, tea and red wine	ZIKV	- Inhibits post-entry processes of viral life cycle - Inhibits viral RNA production and envelope protein synthesis	- Time-of-addition and time-of-removal assays, qRT-PCR, and western blot	[107]
13	Quercetin 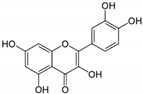	*Bauhinia longifolia* leaves, tea, apple, onion and tomato	DEN-2	- Inhibits intracellular viral replication but not viral attachment and entry processes	- Foci forming unit reduction assay, qRT-PCR	[108]
			MAYV *	- Virucidal activity observed	- Virus yield inhibition assay	[82]
14	Quercetagetin 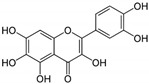	Leaves of *Eriocaulon* species.	CHIKV	- Inhibits viral attachment to host cells - Has potent neutralizing effect against extracellular CHIKV particles	- Time-of-addition assay, inactivation assay	[95]
15	Quinine 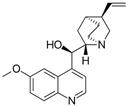	Cinchona tree	DEN-1 to -4	- Inhibits viral replication by reducing DENV RNA and viral protein synthesis	- Virus internalization assay, focus-forming unit (FFU) assay real time RT-PCR, western blot	[109]
16	Resveratrol 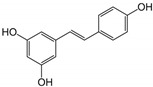	Grapes and peanuts	ZIKV	- Inhibits viral attachment to host cells - Virucidal activity observed	- Pre-treatment and post-treatment assay, focus-forming assay, qRT-PCR, anti-adsorption and virus internalization inhibition assay	[110]
17	ST081006 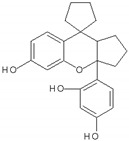	Synthetic flavonoid from flavonoid-derivative library	DEN-1 to -4	- Affects synthesis of both viral protein and RNA	- Pre-treatment, time-of-addition, and time-of-removal assays, qRT-PCR and western blot	[111]

* denotes arbovirus not currently circulating in Asia.

**Table 3:** List of plant extracts that exhibited antiviral activity against arboviruses

S/N	Plant Extract (active compound)	Source	Virus(es) Affected	Mode of Inhibition	Assay Used	Ref.
1	*Aphloia theiformis* extract^	*Aphloia theiformis*	DEN-1 to -4	- Inhibits viral attachment to host cells	- Foci-forming immunodetection assay, virus inactivation assay	[81]
			ZIKV	- Inhibits viral attachment to host cells		
2	Mushroom extracts^	*L. rhinocerotis*, *P. giganteus*, *H. erinaceus* and *S. commune*	DEN-2	- Inhibits viral attachment and entry to host cells	- Time-of-addition studies, plaque reduction assay, and RT-qPCR	[112]
3	*Psiloxylon mauritianum* extract^	Aerial parts of *Psiloxylon mauritianum*	DEN-1 to -4	- Inhibits viral attachment to host cells	- Time-of-addition assay	[113]
			ZIKV	- Inhibits viral attachment to host cells		
4	Silymarin complex (Silybin)	Seeds of *Silybum marianum* (Milk thistle)	CHIKV	- In vitro antiviral activity observed - Inhibits post-entry stages of viral replication cycle	- Time-of-addition and time-of-removal studies, qRT-PCR and western blot	[114]
			MAYV *	- In vitro antiviral activity observed - Protective effect against virus-induced oxidative stress	- – Plaque reduction assay, measuring reactive oxygen species production of MAYV infected cells before and after silymarin treatment	[115]

* denotes arbovirus not currently circulating in Asia. ^ denotes active compound not reported in literature.

**Table 4:** Overview of the natural compounds which can inhibit the different arboviruses

Type of Inhibitors	Antiviral Natural Compounds ^1^
DENV inhibitors	Baicalein, curcumin, delphinidin, EGCG, honokiol, naringenin, quercetin, quinine, and ST081006
JEV inhibitors	Baicalein, curcumin, and kaempferol
WNV inhibitors	Delphinidin and EGCG
ZIKV inhibitors	Curcumin, delphinidin, EGCG, isoquercitrin, pinocembrin, and resveratrol
CHIKV inhibitors	Baicalein, curcumin, EGCG, fisetin, harringtonine, and quercetagetin
MAYV inhibitor	Quercetin

^1^ Refer to Table 2 for references for the respective compounds.

**Table 5:** Overview of the plant extracts which can inhibit the different arboviruses

Type of Inhibitors	Antiviral Plant Extracts ^1^
DENV inhibitors	*Aphloia theiformis* extract, mushroom extracts and *Psiloxylon mauritianum* extract
ZIKV inhibitors	*Aphloia theiformis* extract and *Psiloxylon mauritianum* extract
CHIKV inhibitors	Silymarin complex
MAYV inhibitor	Silymarin complex

^1^ Refer to Table 3 for references for the respective compounds.
